# Membrane Topology and Predicted RNA-Binding Function of the ‘Early Responsive to Dehydration (ERD4)’ Plant Protein

**DOI:** 10.1371/journal.pone.0032658

**Published:** 2012-03-14

**Authors:** Archana Rai, Penna Suprasanna, Stanislaus F. D'Souza, Vinay Kumar

**Affiliations:** 1 Nuclear Agricultural & Biotechnology Division, Bhabha Atomic Research Centre, Mumbai, India; 2 High Pressure & Synchrotron Radiation Physics Division, Bhabha Atomic Research Centre, Mumbai, India; University of Cyprus, Cyprus

## Abstract

Functional annotation of uncharacterized genes is the main focus of computational methods in the post genomic era. These tools search for similarity between proteins on the premise that those sharing sequence or structural motifs usually perform related functions, and are thus particularly useful for membrane proteins. Early responsive to dehydration (*ERD*) genes are rapidly induced in response to dehydration stress in a variety of plant species. In the present work we characterized function of *Brassica juncea ERD4* gene using computational approaches. The ERD4 protein of unknown function possesses ubiquitous DUF221 domain (residues 312–634) and is conserved in all plant species. We suggest that the protein is localized in chloroplast membrane with at least nine transmembrane helices. We detected a globular domain of 165 amino acid residues (183–347) in plant ERD4 proteins and expect this to be posited inside the chloroplast. The structural-functional annotation of the globular domain was arrived at using fold recognition methods, which suggested in its sequence presence of two tandem RNA-recognition motif (RRM) domains each folded into βαββαβ topology. The structure based sequence alignment with the known RNA-binding proteins revealed conservation of two non-canonical ribonucleoprotein sub-motifs in both the putative RNA-recognition domains of the ERD4 protein. The function of highly conserved ERD4 protein may thus be associated with its RNA-binding ability during the stress response. This is the first functional annotation of ERD4 family of proteins that can be useful in designing experiments to unravel crucial aspects of stress tolerance mechanism.

## Introduction

Dehydration is one of the most common environmental stresses that soil plants are exposed to affecting their growth and development through alternation in metabolism and gene expression [1]. Plants induce a large number of genes under water stress, which can be divided into two categories based on the time of induction: responsive to dehydration and early responsive to dehydration [2], [3]. However, the exact function of many stress tolerance associated gene products is still unknown and the encoded proteins have been grouped as hypothetical domains of uncharacterized functions (DUF).

Early responsive to dehydration (*ERD*) genes are rapidly induced to respond to dehydration and various other abiotic stresses. A total of sixteen complementary DNAs for early response to dehydration genes have been isolated from 1 hour dehydrated *Arabidopsis thaliana* which included the *ERD4* gene [4]. The *ERD4* encoded protein (ERD4) has been validated as gene product in *A. thaliana* [2], [4]–[5], in *Zea Mays* [6], and in *Saccharum officinarum* [7]. However, due to lack of information of its structure and function, ERD4 has been classified as belonging to DUF221 protein family (Pfam, PF02714) found in a family of hypothetical transmembrane proteins, none of which have any known function. Also, the organelle localization of the ERD4 protein has been debated in plasma, mitochondria and chloroplast membranes.

The identification of geometric relationships between protein structures, by the use of structural alignment methods, offers a powerful approach in identifying structural and functional relationships between highly divergent proteins [8]. It is well established that proteins evolve partly through rearrangements of larger fragments, typically domains, and nature of these fragments determine biological function of proteins [9]. The analysis of proteins at individual domain levels can facilitate functional annotation of uncharacterized genes and proteins [10]–[12]. Recently, function of a large number of proteins of DUF families has been proposed based on the structural homology of experimentally determined structures to functionally annotated proteins [13]. The functional domains can also be identified reliably by computational analysis such as prediction of the secondary structure, transmembrane segments, and by fold-recognition [14], [15]. An atomic model of the identified domain can further be obtained from the sequence alone by identifying homologs using sequence-sequence comparison or by fold assignment using structure-sequence alignment [16], [17]. With the available computational tools, it is also possible to identify residues involved in the biological function based on the structure-structure comparison. The utility of these approaches can be extended for predicted structural models of uncharacterized proteins enabling functional annotation of related proteins. Such a strategy is particularly useful for membrane proteins as their experimental structure-function determination is a difficult task.

We investigated the function of the *Brassica juncea* ERD4 protein using a combination of advanced sequence profile searches and structure prediction bioinformatics approaches like fold recognition and comparative modeling. We found a globular domain in ERD4 sequence. The globular domain resides inside the chloroplast and belongs to RNA-binding protein superfamily. The domain has two RNA-recognition motifs, typical of RNA-binding proteins. Also, conservation of the RNA-binding residues was observed by structure comparison methods.We suggest that ERD4 has a role in post transcriptional gene regulation. The bioinformatics analyses presented here offers the first hypothesis about the function of the ERD4 family of proteins.

## Results

### Sequence and phylogenetic analyses

**Figure 1:**
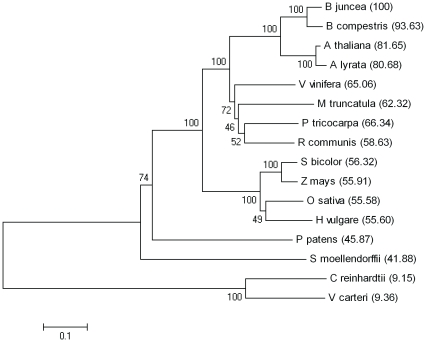
Evolutionary relationship among ERD4 homologs. Evolutionary relationship was inferred using the Neighbor-Joining method in MEGA4 software. The percentage of replicate trees in which the associated taxa clustered together in the bootstrap test (100 replicates) is shown next to the branches. The tree is drawn to scale with branch lengths in the same units as those of the evolutionary distances used to infer the phylogenetic tree. The evolutionary distances are in the units of the number of amino acid substitutions per site. Also shown in brackets are the pair-wise percentage identity between *B. juncea* ERD4 and other plant proteins, including green algae.

The 3291 bp long nucleotide sequence of *B. juncea ERD4* gene structure study suggests that this gene codes for mRNA of length 2172 (6 exons and 5 introns) which encodes 723 amino acids long protein (UniProtKB, A9LIW2). The homologs of *B. juncea* ERD4 protein were identified in various plant lineages, for instance in bryophyta (*Physcomitrella patens*), in traceaophyta (*Selaginella moellendorffii*), in euphylophyta (*O. sativa*, *A. thaliana*). The protein was found to be conserved in all the plants for which proteome data was available (Fig. 1). Phylogenetic tree of plant ERD4 homologs showed four distinct clades and the evolution pattern of this gene followed the lineages evolution (Fig. 1). The presence of both putative RNA-binding and DUF221 domains, a characteristic of plant ERD4 proteins, was also detected in unicellular (*C. reinhardtii*) and multicellular (*V. carteri*) green algae genomes by iterative PSI-BLAST search. The algal proteins, however, consists of 1746 and 1172 residues, respectively (UniProtKB, A8HT24 and D8TSA1). However, homolog of plant ERD4, possessing both the RRM and DUF221 domains, were not detected in bacteria (including cyanobacteria) and archae. Counter intuitively, ERD4-like proteins were detected in unicellular non-photosynthetic eukaryotes like *Dictyostelium fasciculatum* (slime mould) and colonial flagellates like *Choanoflagellates*. These proteins showed 24.5% (52.7%) and 19% (40%) sequence identity (similarity), respectively, with *B. juncea* ERD4 protein over the complete length. We also detected proteins possessing both the RNA-binding and DUF221 domains in fungi including many plant pathogens (for instance, in *Phytophthora sojae*) and in animals. A *Homo sapien* ortholog of the identified animal proteins has recently been characterized as “transmembrane protein 63A” (UniProt/KB, O94886; TM63A_human). The human protein consists of 807 amino acid residues and shows 24% (41%) sequence identity (similarity) over 608 residues with *B. juncea* ERD4 protein (Fig. S1).

The motif scanning (motif_scan) and domain detection tools (Pfam, DOUTfinder and SMART) detected presence of DUF221 domain (residues 312–634) in the ERD4 sequence with very high confidence (E-value, 7e-146). The DUF221 domain is found in a family of hypothetical transmembrane proteins none of which have any known function. This domain has been identified in all forms of eukaryotic organisms and has been observed in different domain architectures in combination with a variety of other functional domains like PIWI, phosphate metabolism protein etc. The DOUTfinder also identified potential similarity with eukaryotic RNA-recognition motif with 10% false-positive rate. The biological relevance of this was, however, not clear owing to highly distant sequence similarity as suggested by poor D-score of 163 [18].

### Transmembrane topology and localization

**Figure 2:**
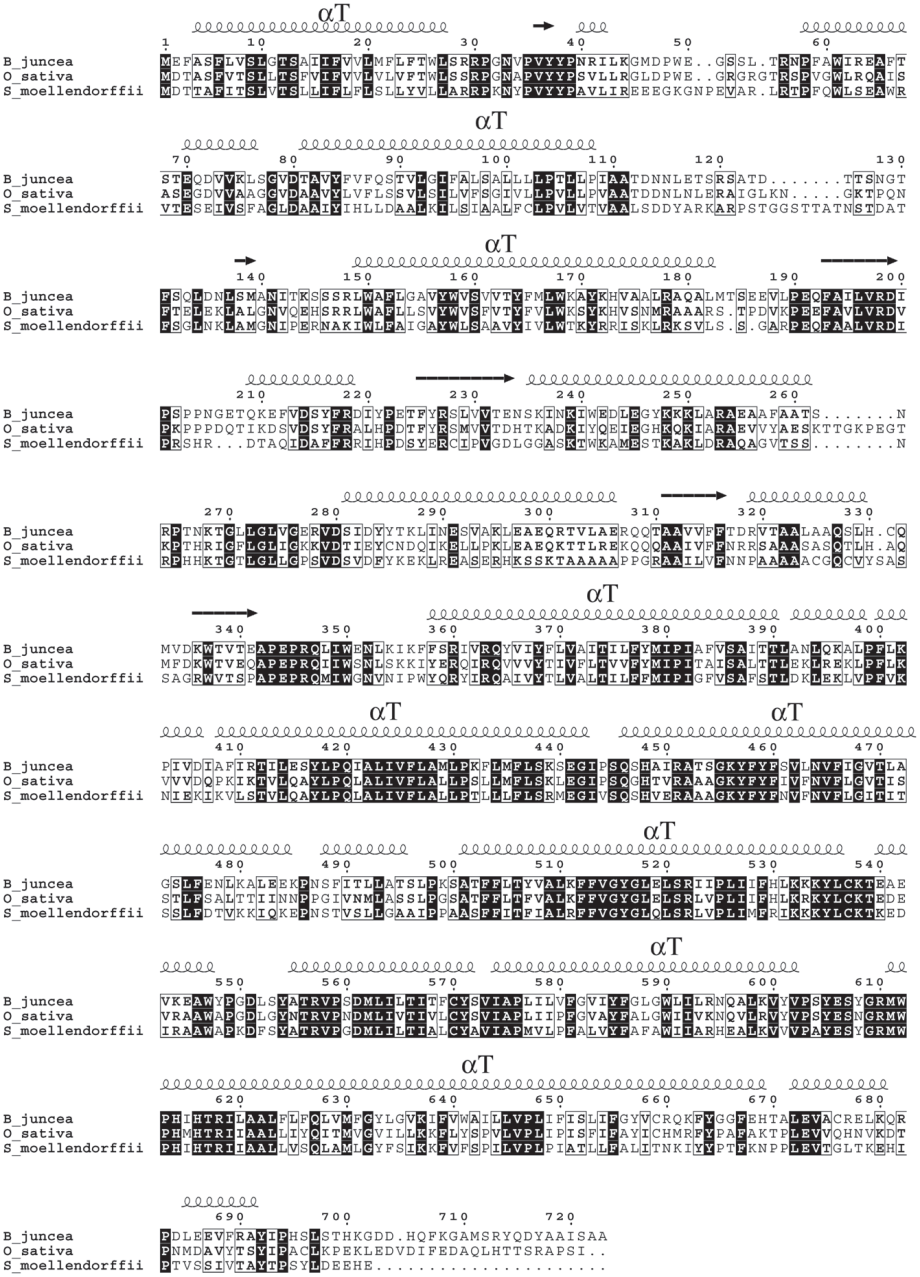
Multiple sequence alignment of plant ERD4 sequences. The alignment of all available plant ERD4 sequences was achieved using PROMALS3D [42] and only three diverse sequences are shown here. Also shown is the consensus secondary structure predicted by PsiPred; helices are shown as coils and strands are shown as arrows. The nine transmembrane helices are marked as αT. The strictly conserved residues in all the plant ERD4 sequences are shaded, while similar residues are boxed. The residues numbering is of the full-length *B. juncea* ERD4 protein. The figure was prepared with EsPript suite [64].

Transmembrane helices in the ERD4 sequence were identified using several web-servers *albeit* with some differences. The number of identified helices varied from 9 to 11 and the suggested starting- and end- points for predicted transmembrane segments also differed. Based on high-confidence predictions from different servers, nine transmembrane helices belonging to the sequence regions of 6–26, 90–111, 149–167, 365–385, 419–437, 457–476, 501–531, 573–593 and 638–659 were identified (Fig. 2). The identification of the transmembrane helices was consistent with the predicted secondary structure which suggested that the ERD4 protein is mainly helical with 64.3, 5.4 and 30.3% residues in helix, extended and coil structures, respectively. Interestingly, all the transmembrane prediction tools showed that a long polypeptide segment (residues 170–360) did not possess transmembrane helices (non-transmembrane segment). A globular domain was subsequently detected in this segment.

Maximum probability of localization of this protein was predicted in plasma membrane (with score of 10) followed by chloroplast (score 2) using Wolf PSORT tool. The YLoc tool, however, suggested its presence in chloroplast with 53.9% probability and a small confidence (0.27). The TargetP server predicted this protein to be a secretory protein with high confidence (score 0.92). The analysis of *B. juncea* ERD4 by the ambiguous targeting predictor (ATP) suggested a score of 0.39, which weakly suggested dual targeting of the ERD4 protein. The analysis of ERD4 orthologs by the ambiguous targeting predictor, however, suggested wide variations in the confidence score (Table 1) with a low score of 0.19 for some ERD4 proteins that clearly indicated localization of ERD4 in only one compartment. Although the used predictors failed to identify unambiguously the chloroplastic localization of the ERD4 protein, its localization in chloroplast membrane has been shown experimentally in Arabidopsis [19].

**Table 1:** Prediction scores for dual organelle targeting of plant ERD4 proteins assessed by ambiguous targeting predictor (APS).

Plant species	Accession code	Source	APS prediction score
*Brassica juncea*	A9LIW2	UniProtKB	0.39122
*Brassica campestris*	A8IXK5	UniProtKB	0.39122
*Arabidopsis thaliana*	Q9C8G5	UniProtKB	0.19248
*Arabidopsis lyrata*	D7KET4	UniProtKB	0.19248
*Populus tricocarpa*	B9GJG0	UniProtKB	0.39122
*Sorghum bicolor*	C5X9J3	UniProtKB	0.47346
*Vitis vinifera*	F6HLU8	UniProtKB	0.30121
*Oryza sativa*	Q6ZLQ0	UniProtKB	0.34804
*Zea mays*	B0FSL2	UniProtKB	0.47346
*Medicago truncatula*	AES64128	GenBank	0.20827
*Ricinus communis*	B9SY14	UniProtKB	0.39122
*Hordeum vulgare*	F2DDW1	UniProtKB	0.34804
*Physcomitrella patens*	A9TEC4	UniProtKB	0.41759
*Selagilella moellendorffii*	D8STJ2	UniProtKB	0.29168
*Chlamydomomas reinhardtii*	A8HT24	UniProtKB	0.49063
*Volvox carteri*	D8TSA1	UniProtKB	0.21542

**Figure 3:**
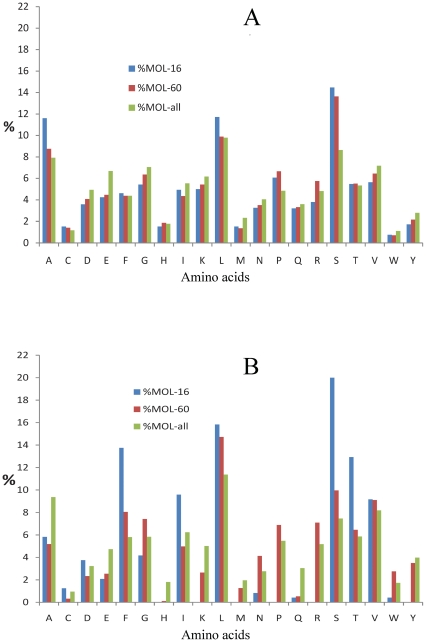
Amino acid composition of presequences. Analysis of the amino acid composition of the N-terminal sixteen residues (%MOL-16), N-terminal sixty residues (%MOL-60) and full-length proteins (%MOL-all) (A) analysis of the 123 chloroplast envelope proteins of *A. thaliana* (B) analysis of plant ERD4 orthologs.

It has been earlier shown that N-terminal sixty residues contain signal sequence for chloroplastic localization, sixteen of which could be used to discriminate between mitochondrial and chloroplastic localization [20]. In order to get detailed information on the amino acid composition of presequences for chloroplast envelope targeting, we analyzed experimentally validated chloroplastic envelope proteins of *A. thaliana*. An overall amino acid composition and N-terminal sequence logo plots of the 123 selected proteins (ENV dataset) from Arabidopsis proteome [19] were analyzed. The positional abundance of amino acids in sequence logos showed abundance of Ser residues and underrepresentation of Arg residues in the ENV dataset. However, no clear position-specific pattern was observed in sequence logo plots. Similar trends have earlier been observed for the total chloroplast proteins, including stroma proteins [20], [21]. The amino acid composition analysis also showed much higher abundance of Ser, Ala and Leu residues in the N-terminal sixteen residues as compared to the full-length proteins (Fig. 3A). Also, the percentage of Arg residues in the N-terminal sixteen residues was observed to be lower than that observed in full-length or N-terminal sixty residues. The analysis of the N-terminal sixteen residues of the ERD4 orthologs also showed similar trends; higher abundance of potentially hydroxylated Ser/Thr residues and of hydrophobic Phe/Ile residues. The N-terminal sixteen residues also showed high differences in the abundance of Arg and Lys residues, as compared to the N-terminal sixty and overall composition of these proteins. These positively charged residues are underrepresented in the N-terminal sixteen residues of the ERD4 orthologs (Fig. 3B). The lower abundance of Arg and Lys residues in the N-terminal sixteen residues of chloroplast proteins, compared to mitochondrial proteins, has been earlier observed by Bhushan et al. [20]. The low percentages of the positively charged Arg/Lys residues and significantly higher percentage of Ser residues in the N-terminal sixteen residues of ERD4 proteins thus corroborated experimental determination of the ERD4 protein in *A. thaliana* chloroplast envelope proteome.

**Figure 4:**
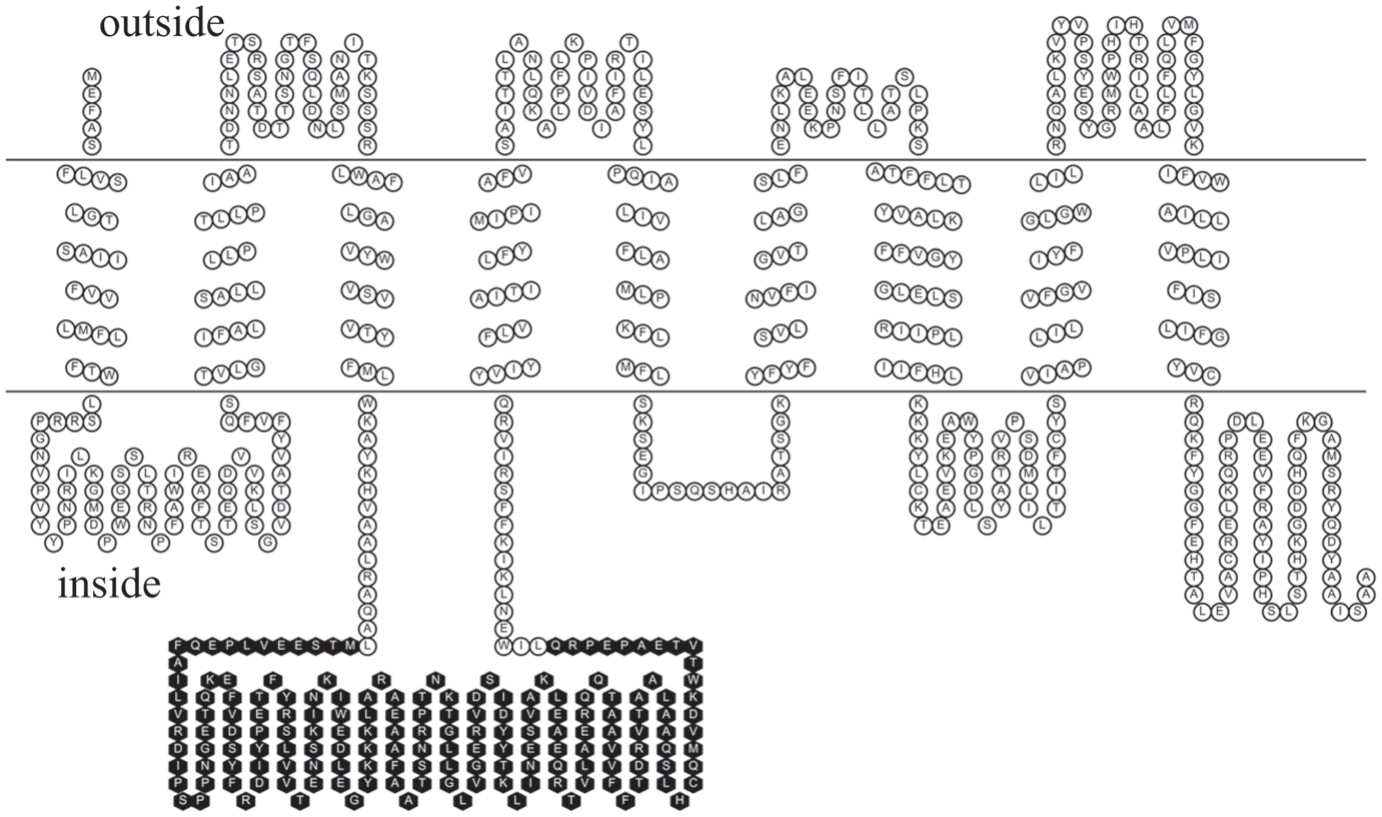
The topology of the *B. juncea* ERD4 protein. The toplogy was drawn using TOPO2 tools. The nine transmembrane helices are shown. Also, shown (filled hexagons) is the globular domain containing RNA-recognition domains. The globular domain is suggested to reside inside the chloroplast.

The inside or outside localization of the non-transmembrane fragment (inside or outside the chloroplast membrane) depended upon the orientation of N-terminal transmembrane helix. While MEMSAT and TMpred showed its placement inside the membrane, several other tools like HMMTOP, TMHMM, TMMod predicted its presence outside the membrane. These predictions resulted in two distinct membrane topologies and the ambiguity was resolved using frequency of the positively charged residues in both the possible topologies. It was concluded that N-terminus of ERD4 was outside the membrane as nearly 79% of the positively charged residues were observed to reside on inside loops. The corresponding transmembrane topology model revealed presence of the non-transmembrane segment (residues 170–360) inside the chloroplast (Fig. 4). The predicted secondary structure showed nearly 47% residues in helix, 12.6% residues in β-strand and 40.4% residues in the coil structure, respectively, in this segment.

### Structural analysis of the globular domain

A BLAST search with the amino-acid sequence did not reveal any close homologue in the database of known protein structures (PDB). This is not unusual as sequence comparison methods cannot reliably detect evolutionary relationship between highly divergent proteins. The structural fold of the ERD4 domain was then found by fold-recognition methods, which use sequence-structure alignment. This method allows detection of remote homologies beyond the detection limits of other sequence comparison methods. The input for fold-recognition was *B. juncea* ERD4 sequence from which generated profile was compared to sequence profiles of proteins and domains of known structures. The search for ERD4 protein fold using fold-recognition meta-server suggested structural homology of about 165 amino acid residues (183–347) with the known RNA-binding globular proteins. Interestingly, all the best hits identified by the 3D-jury from the meta-server were RNA-binding proteins possessing two well known RNA-recognition motifs (RRM) (Table 2). The residues 183–347 of the ERD4 sequence were thus expected to adopt a globular fold with structural similarity with RNA-binding proteins

**Table 2:** The best five structural models predicted for the ERD4 globular domain by the fold-recognition servers and their ranking by 3D-Jury method.

Model (1)	3D-Jury score(JScore)	Scop [63]		Percentage identity/similarity with *B. juncea* ERD4 globular domain
		Classification	Superfamily	
2krr _A	55.3	54928	RNA-binding domain	9.6/27.1
2dhs_A	54.0	54928	RNA-binding domain	12.1/36.4
1cvj_A	48.0	54928	RNA-binding domain	7.8/26.1
2g4b_A	41.0	54928	RNA-binding domain	11.5/30.9
3md3_A	39.7	54928	RNA-binding domain	10.3/32.1

(1) PDB identifier code.

**Figure 5:**
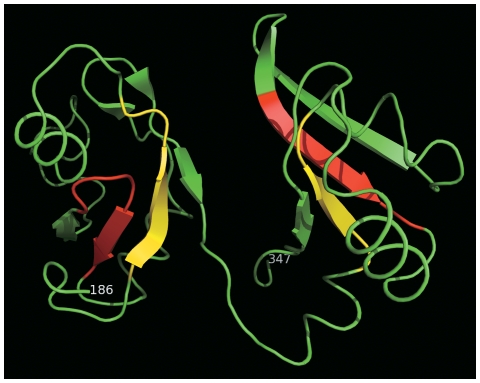
Ribbon model of the putative RNA-binding globular domain. The ribbon model was constructed by comparative homology approaches. The fold of the domain was identified by fold-prediction meta-server. Due to low pair-wise sequence identity of nearly 10% between the query and identified template, the derived atomic coordinates for the ERD4 globular domain were expected to be of low-resolution. The two ribonucleoprotein motifs (RNP1 and RNP2) in each of the RNA-recognition domains are shown in red and yellow, respectively. The figure was prepared by PyMol (http://www.pymol.org/).

The 3D structural models of the globular domain were constructed using the solution structure of the RBD1,2 domains from human nucleolin (PDB code, 2KRR; Jscore, 55.3) and using X-ray crystal structure of the poly(a)-binding protein in complex with polyadenylate RNA (PDB code, 1CVJ; Jscore, 48) as templates. Given the high divergence between ERD4 globular domain and the RNA-recognition proteins used for constructing the theoretical models with pair-wise sequence identity of about 10% (Table 2), we would expect the general atomic resolution of the theoretical model to be low (>3 Å). However, all the structural neighbors of the ERD4 globular domain were found by DALI program [22] to belong to RNA-binding domain superfamily. The computationally constructed structural models for the ERD4 chloroplastic domain clearly showed the presence of two tandem RNA-recognition motifs, each having βαββαβ topology (Fig. 5). The two RRM domains are composed of amino acid residues 183–269 (RRM1) and 273–347 (RRM2) respectively, and are joined by an interdomain linker peptide. The interdomain linker peptide is a typical characteristic of known RNA-binding proteins with multiple RRM domains [23]. The two RRM domains could be flexibly tethered via the linker peptide. Analogous to the well characterized RNA-binding proteins, the β-sheets of the two RNA-binding domains of ERD4 face each other and RNA substrates could bind in the cleft.

**Figure 6:**
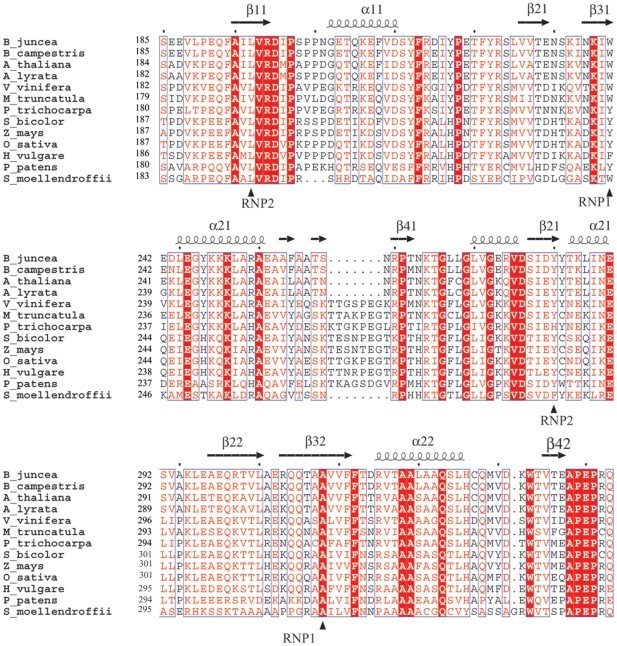
Multiple sequence alignment of the ERD4 globular domain. The alignment was generated by ClustalW. The two RNA-recognition domains are composed of amino acid residues 183–269 (RRM1) and 273–347 (RRM2), respectively. The two ribonucleoprotein motifs of each RRM domain are marked as RNP1 and RNP2. The suggested RNA-interacting residues are marked with filled triangle (▴). The secondary structure elements of each RRM domain in the theoretical structural model are also shown. The strictly conserved residues in all the plant ERD4 sequences are shaded, while similar residues are boxed. The residues numbering is of the full-length ERD4 proteins.

The two RNA-recognition domains of ERD4 were individually superposed onto the known RNA-binding domains of sex lethal protein (PDB code, 1B7F) and adenosine-uridine (AU)-rich binding Hu protein (PDB code, 1FXL). These proteins had similar number of amino acids as ERD4 globular domain but differed significantly from the latter (DaliLite Z-scores for ERD4/1B7F and ERD4/1FXL pairs were 5.8 and 5.5, respectively) and thus formed highly diverse pairs. Additionally, these structures had been refined to high precision against single crystal diffraction data and coordinates of protein-RNA complexes were available, which could hint RNA-binding mode in the ERD4 protein (Fig. S2). The structural alignment showed the presence of two non-canonical ribonucleoprotein sub-motifs (RNP1 and RNP2) in both the ERD4 domains (Fig. 6). One of the ribonucleoprotein sub-motifs (RNP2) resides on the first β-strand, while residues from third β-strand contribute towards RNP1. The putative RNP sub-motifs of RRM1 are 195-ILVRDI-200 (RNP2) and 237-INKIWEDL-244 (RNP1) and those of RRM2 are 283-DYYTKL-288 (RNP2) and 307-RQQTAAVVF-315 (RNP1). In the multiple sequence alignment of ERD4 orthologs, the RRM1 domain has conserved hydrophobic (Leu/Val) at position-2 of the RNP2 and aromatic (Trp/Tyr) at position-5 in RNP1 (Fig. 6). Also, Tyr/His and Ala are conserved in RNP2 position-2 and RNP1 position-5, respectively, in the RRM2 domain. A positively charged amino acid residue (Arg/Lys) was also found in most of the plant ERD4 proteins at RNP1 position-1 of RRM2. In addition to the â-strands, the loops β1/α1 (connecting β1 and α1 elements), β2/β3 and α2/β4 have also been observed in RNA-binding proteins to interact with nucleic acid substrates [23]. Most of these residues are conserved in ERD4 orthologs (Fig. 6). Interestingly *B. juncea* Pro-201, residing on the loop β1/α1, is strictly conserved in all the plant ERD4 proteins. This position is occupied by Pro/Ser residues in majority of RNA-binding domains identified in NCBI conserved domains database CD00590 [24].

## Discussion

A close homolog of *Brassica juncea* ERD4 protein was detected in all plant species indicating conservation of the protein in plantae kingdom. Phylogenetic relationship of this gene showed similar pattern of divergence as different plant lineages have evolved, emphasizing that *ERD4* gene has been essentially maintained during the course of plant evolution (Fig. 1).

A consensus assignment using high confidence prediction scores suggested that ERD4 is a transmembrane protein with at least nine transmembrane helices in the ERD4 sequence (Fig. 2). Its localization in different plant organelle has been subject of intense discussion recently. Its localization in the chloroplast membrane was earlier suggested from the Arabidopsis chloroplast envelope proteome analysis [5], [19], while Alexandersson et al. [25] identified its location in plasma membrane of *Arabidopsis thaliana* that could have been due to organelle contamination [26]. Further, mitochondrial and plastid dual targeting of *A. thaliana* ERD4 was suggested [27]. The analysis of homologous plant ERD4 sequences was used here for confirming its organelle localization on the premise that localization signatures must be strictly conserved in all the plant ERD4 sequences. The analysis of ERD4 orthologs by the ambiguous targeting predictor suggested wide variations in the confidence score; a low score of 0.19 for a number of ERD4 orthologs (Table 1) clearly indicated its localization in only one compartment. Its presence in chloroplast membrane, however, was inferred on the basis of higher abundance of Ser/Thr and underrepresentation of Arg/Lys residues in the N-terminal sixteen residues of ERD4 orthologs, as also observed earlier for the chloroplast proteins [20]. We also found marked increase in percentage of hydrophobic Ala/Leu residues in the N-terminal sixteen residues for chloroplast envelope proteins of *A. thaliana*. Similar high percentage of hydrophobic Phe/Ile residues was observed in the N-terminal sixteen residues of ERD4 orthologs (Fig. 3B). Taken together these data support the experimental finding of its localization in chloroplast membrane. The presence of ERD4 in the chloroplast is also consistent with predominance localization of the organelle stress response proteins in chloroplast as noted recently by Taylor et al. [28]. The detection of ERD4-like protein in uni- and multicellular green algae provides further credence to our suggested chloroplastic localization of the ERD4 protein, as all plastids derive from a single endosymbiosis and after plastid acquisition only photosynthetic eukaryotes diverged into glaucocystophytes, rhodophytes, and viridiplantae lineages [29]–[31]. However, ERD4-like protein was not detected in cyanobacteria. Previous findings have also reported that plant proteins encoded by genes of cynobacterial origin are not, as a rule, targeted to chloroplast, whereas many non-cynobacterial proteins can be targeted to plastids [32].

A transmembrane DUF221 domain (312–634) and a globular domain (183–347) were identified in the Brassica ERD4 sequence. The DUF221 domain has been identified in all forms of eukaryotic organisms and has been observed in nearly 23 different domain architectures in combination with a variety of other functional domains like Dnaj, UBQ, VWD etc. The existence of structural domain, with a common function, in combination with variety of other domains has been known to be responsible for evolution of protein repertoire [33]. The DUF221 domain has no other known function, except for membrane integration. It is likely that biological function of the ERD4 protein is attributed mainly to the globular domain, and DUF221 helps in localization of the functional (globular) domain. The deduced topology, based on the positive-inside rule, reveals that the globular domain resides inside the chloroplast (Fig. 4). The smaller loops reside on outside the membrane confirming also to the observation that periplasmic loops are short possibly because of difficult translocation of intermediate-length loops [34].

The structural analysis is known to reveal the distant evolutionary links that could provide the first hypothesis about biological function of the uncharacterized domains [13]. The tertiary structure of the ERD4 chloroplastic globular domain was predicted by fold-prediction algorithms that suggested presence of two RNA-recognition motifsin its sequence. Each of the RRM was predicted to adopt βαββαβ topology (Fig. 5,6). The fold of the ERD4 globular domain was found to be shared only by RNA-binding domains, as observed in the search for structural neighbors with DALI programs. Structural and sequence comparison with the known RNA-binding proteins showed the presence of RNP1 and RNP2 ribonucleoprotein sub-motifs in both the identified RNA-recognition motifs of ERD4. The four RNP's in two RRM domains reside on the â-strands creating a RNA binding cleft (Fig. 5). A hydrophobic and an aromatic amino acid residue at 2^nd^ and 5^th^ positions of RNP2 and RNP1, respectively, were conserved in RNA-binding proteins and ERD4 homologs (Fig. 6). These residues stack against the two bases of substrate RNA in the known RNA-binding proteins. The 1^st^ position of RNP1 in RRM2 of ERD4 was also found to be conserved as positively charged amino acid that could neutralize the negatively charged phosphodiester group [35]. In most of the RRM-RNA complex structures only one to three of these contacts are observed with two stacking interactions involving RNP2 position-2 and RNP1 position-5 observed most frequently [36]. The orthologs of TM63A_human protein identified by BLAST search due to its sequence similarity with plant ERD4 proteins, however, do not show strict conservation in the residues corresponding to the proposed RNA-binding domain of ERD4 (Fig. S1). In contrast to RNA-binding ability, polypeptides that recognize protein substrates, and not RNA, have only one RRM domain. The combination of two or more RNA-recognition motifs, as observed in ERD4 sequences, often results in dramatically increased RNA-binding affinity [23], [37].

The RNA binding domain carrying RNP signature sequences is a highly abundant domain in eukaryotes. This domain has been found in a variety of heterogeneous nuclear ribonucleoproteins (hnRNPs), proteins implicated in regulation of alternative splicing, and protein components of small nuclear ribonucleoproteins (snRNPs), and is involved in post-transcriptional gene expression processes including mRNA and rRNA processing, RNA export, and RNA stability. The domain binds a variable number of nucleotides, ranging from two to eight. It is, however, known that despite using the same β-sheet surface to bind RNA, each protein achieves sequence-specificity slightly differently [23].The conservation of two tandem RNA-recognition motifs and the substrate binding residues suggests that globular domain of ERD4 protein may be RNA-binding competent.The ERD4 protein can participate in mRNA metabolism such as sequestering and protecting mRNAs during conditions of limiting transcription. In plants, the RNA-binding proteins may modulate ABA signaling through the alteration of mRNA processing events such as splicing, processing, nuclear export, transcript stability and RNA degradation [38]. Also, induction of ERD4 could influence the membrane fluidity as its DUF221 domain is expected to be integrated in the chloroplast membrane. It hence assumes significance to study functionally important residues and domains that are critical for ERD4 activity in response to various environmental conditions. We also suggest from the analysis that ERD4 proteins may be characterized by the presence of both RRM and DUF221 domains and not by DUF221 domain alone as is the current practice in putative annotations in the sequence databases.

### Conclusion

The ERD4 protein is a transmembrane protein whose role has been identified in abiotic stress amelioration in plants. Based on sequence analysis, we expect its location in chloroplast membrane. A globular chloroplastic domain was detected in its sequence that is suggested to possess two tandem RNA-recognition motifs. Detection of RNA binding residues in the globular domain further suggests that the biological function of ERD4 may be associated with its RNA-binding ability. Understanding of structure-function of *ERD4* gene product may help in understanding plant stress response and in enhancing plant tolerance to environmental stresses.

## Materials and Methods

### Sequence based analyses

The *Brassica juncea ERD4* gene sequence was obtained from the Genbank (accession number: EU126607). Gene structure study was performed using popular gene finding pipeline (FGENESH at www.softberry.com). The homologs of *B. juncea* ERD4 protein sharing better than 40% sequence identity were obtained from UniProt database using FASTA search engine. The search for ERD4 homologs using BLAST search engine was carried out also against the non-redundant protein sequences and against translated individual proteome of *C. reinhardtii*, *C. merolae*, several fungi and cyanobacterial (*Synechococcus* sp. RS9916, *Cyanothece* sp., *Nostoc punctiforme*) genomes. To detect ERD4-like proteins in animals, BLAST search against non-redundant protein sequences of animalia (taxid:33208) kingdom was also carried out. Since complete proteome database for *T. aestivum* is yet not available, the search for its homolog was carried out in Ensembl [39] employing tBLASTn [40] search engine.The search of distantly related genomes or those of unrelated species was constrained for the presence of two tandem RNA-recognition motifs and a DUF221 domain detected in the closely related plant species (for discussion on RRM see Results). Multiple sequence analyses were carried out using clustalW and PROMALS3D tools [41], [42]. The phylogenetic tree was derived from that multiple alignment using Neighbor- Joining method in MEGA4 [43]. Motifs were identified using motif scan tools [44].

### Localization and Topology prediction

The prediction for sub-cellular localization of the *B. juncea* ERD4 protein and its orthologs was done using wolf PSORT [45], YLoc [46], TargetP [47],and ambiguous targeting predictor [28] web-tools. Further a subset consisting of 123 chloroplastic envelope proteins of *A. thaliana* chloroplast proteome [19] was analyzed for chloroplast localization signatures. These proteins were identified from the experimentally validated chloroplast envelope protein dataset, those not showing similarity with ribosomal proteins. Amino acid contents were calculated from the complete protein sequence, and for N-terminal sixteen and sixty amino acid residues of this subset of validated chloroplastic proteins and for plant ERD4 proteins.

Secondary structure of the plant ERD4 orthologs were predicted using PsiPred [48] and Prof (http://www.aber.ac.uk/~phiwww/prof/) suites. The web-versions of nine different topology prediction methods were used to estimate membrane topology of ERD4 and these were: DAS [49], HMMTOP [50], MEMSAT [51], TMHMM [52], TMMod [53], TMpred [54], Toppred [55], Conpred [56] and phobias [57]. Modeling of transmembrane topology was done using TOPO2 (http://www.sacs.ucsf.edu/TOPO-run/topoanal-adv2.pl).

### Prediction of the functional domains and 3D structure

The *B. juncea* ERD4 sequence was subjected to Pfam [58], DOUTfinder [18] and SMART [59] analysis for identification of the known domains and domain architecture. An independent analysis for detecting globular domains of structural-folds similar to the known protein structures was also carried out using structure prediction meta-server (http://bioinfo.pl/meta) accessing various fold-recognition and function prediction methods. A globular domain in ERD4 sequence was detected by the fold-prediction meta-server. The database of known protein structures (Protein Data Bank, PDB) was searched for a structure homologus to the detected globular domain using sequence-sequence comparison search engines. In the absence of any known homologus structure, the tertiary fold of the globular domain was independently predicted using the meta-server. The collected results from fold-prediction servers were screened with 3D-jury [60]. The 3D structural model of the globular domain was constructed with Modeller [61] using sequence-to-structure alignment returned by the meta-server, and RNA-binding domains from human nucleolin (PDB code, 2KRR) and poly(a)-binding protein (PDB code, 1CVJ) as templates. The structural neighbors of the theoretical structural model of the globular domain were identified by the DALI [22] programs.

### Identification of functional residues

The 3D structural model of the identified globular domain was superposed onto the known structures of RNA-binding proteins which possessed RNA-recognition domains. The atomic coordinates of these were obtained from the PDB. The superposition was achieved using DALI programs and Swiss PDBViewer [62]. The amino acid residues of the ERD4 domain, equivalent to the residues interacting with RNA substrates in the known RNA-binding proteins, were identified as putative RNA-binding residues. The conservation of these was verified in the alignment of the amino acid sequences of the identified RRM domains of ERD4 homologs.

## Supporting Information

Figure S1 **Multiple sequence alignment of plant ERD4 and proteins of animalia (taxid:33208) kingdom identified by BLAST.** The alignment of plant ERD4 sequences [*B. juncea* (UniProtKB, A9LIW2) and *A. thaliana* (UniProtKB, Q9C8G5)] and diverse animal sequences [*H. sapiens* (UniprotKB, O94886), *X. laevis* (UniProtKB, Q5PQ13) and *N. vectensis* (UniProt KB, A7S3E8)] was achieved using PROMALS3D [1]. The strictly conserved residues are shaded, while similar residues are boxed. The proposed RNA-binding domain of *B. juncea* ERD4 is marked as RBD. A number of insertion/deletions and poor amino acid conservation in the corresponding domains of animal sequences do not suggest close evolutionary relationship between plant and animal proteins. The figure was prepared with EsPript suite [2].
(TIF)

Figure S2 **Cartoon of RNA-binding domain with bound RNA.** Cartoon of HuD1,2–cfos-11 RNA complex structure [PDB code 1FXL; 3]. The RNA is shown as a stick model (orange). The N- & C- termini of the protein are marked as N and C, respectively. The two RRM domains form a cleft with the RNA bound between the β-sheets surfaces. In several RNA-binding proteins the two RRM domains are flexibly tethered via a linker peptide.
(TIF)
