# Supplementary figures and images for: Membrane Topology and Predicted RNA-Binding Function of the ‘Early Responsive to Dehydration (ERD4)’ Plant Protein
Source: PLoS One. 2012 Mar 14;7(3):e32658. doi: 10.1371/journal.pone.0032658 (PMC3303787; doi:10.1371/journal.pone.0032658)

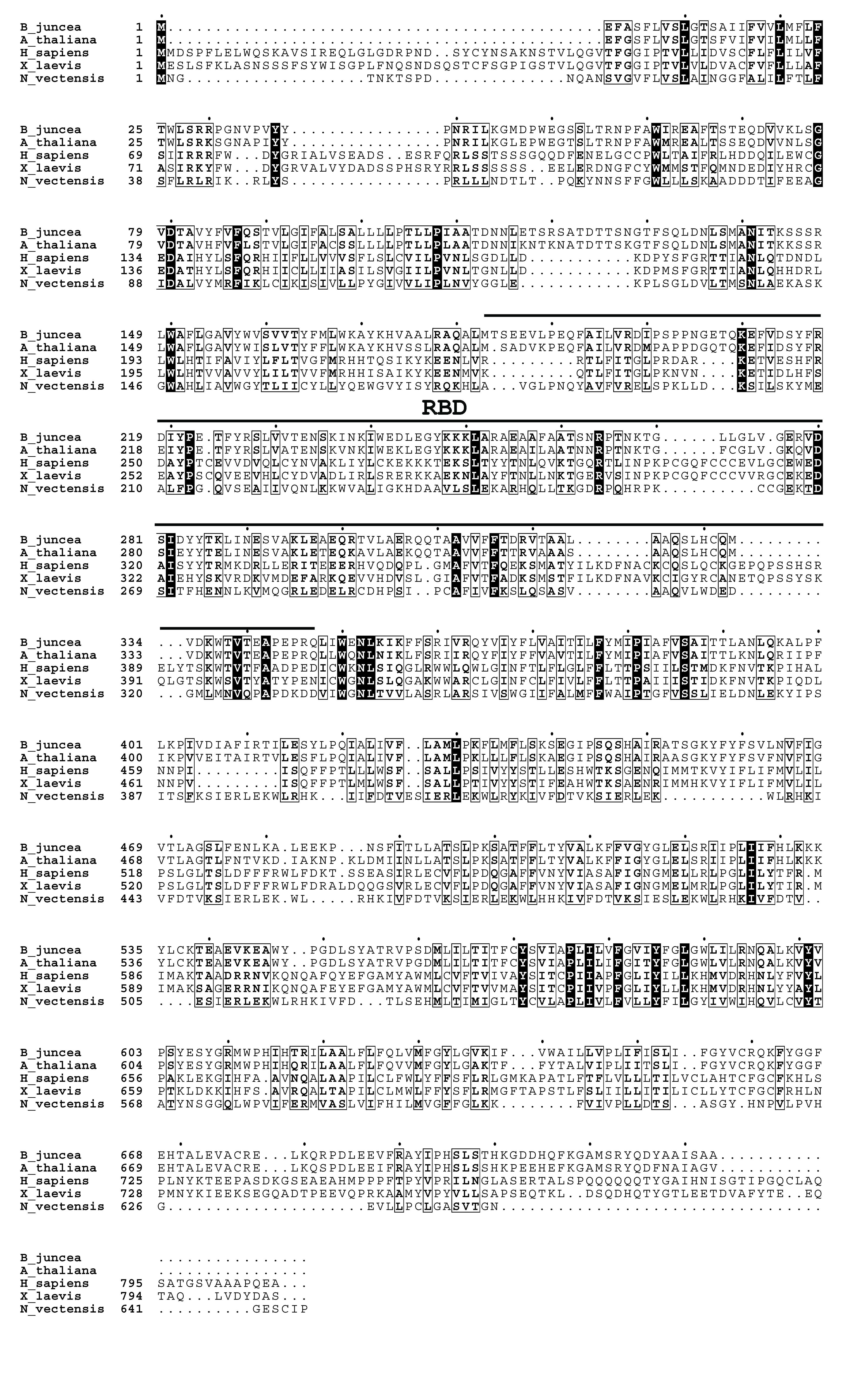

Supplement: Figure S1 — Multiple sequence alignment of plant ERD4 and proteins of animalia (taxid:33208) kingdom identified by BLAST. The alignment of plant ERD4 sequences [B. juncea (UniProtKB, A9LIW2) and A. thaliana (UniProtKB, Q9C8G5)] and diverse animal sequences [H. sapiens (UniprotKB, O94886), X. laevis (UniProtKB, Q5PQ13) and N. vectensis (UniProt KB, A7S3E8)] was achieved using PROMALS3D [1]. The strictly conserved residues are shaded, while similar residues are boxed. The proposed RNA-binding domain of B. juncea ERD4 is marked as RBD. A number of insertion/deletions and poor amino acid conservation in the corresponding domains of animal sequences do not suggest close evolutionary relationship between plant and animal proteins. The figure was prepared with EsPript suite [2]. (TIF) [file pone.0032658.s001.tif]
